# OMI: time for early recognition and management of acute coronary occlusion

**DOI:** 10.1186/s43044-026-00759-y

**Published:** 2026-06-17

**Authors:** Grigorios Avdikos, Stephen W Smith

**Affiliations:** 1 Department of Cardiology, Athens Naval & Veterans Hospital, Athens, Greece; 2 Department of Emergency Medicine, University of Minnesota, Minneapolis, USA; 3 Department of Emergency Medicine, Hennepin County Medical Center, Minneapolis, USA

To the Editor

We read with interest the article by Batubara et al. [1] where they report 3 cases of ST elevation myocardial infarction (STEMI) (-) occlusion myocardial infarction (OMI) that received delayed reperfusion therapy.

In the first case, a 51-year-old man presented with acute myocardial infarction (MI) symptoms with right bundle branch block (RBBB) and left posterior fascicular block (LPFB) on electrocardiogram (ECG). The authors mention that this bifascicular block is an ECG sign of OMI, indicative of proximal left anterior descending coronary artery (LAD) occlusion. However, this ECG is suggestive of postero-lateral OMI: there is ST-segment depression (STD) in leads V2-V3 with hyperacute T-waves in leads I, aVL. This ECG presentation is consistent with left circumflex coronary artery (LCx) OMI. Indeed, coronary angiography revealed diffuse severe stenosis of the proximal LCx with total occlusion of its first branch, which is a large obtuse marginal artery. LAD has diffuse proximal stenosis but is not the culprit artery. Moreover, one can notice the resolution of STD in leads V2-V3 and of hyperacute T-waves in leads I, aVL on the ECG after reperfusion. Thus, it is more likely that in this case, RBBB is not due to acute ischemia and there is not true LPFB but right axis deviation due to lateral wall infarction.

In the second case, a patient presented with Killip class II acute MI. ECG shows sinus tachycardia, RBBB with left anterior fascicular block (LAFB), ST-segment elevation (STE) in leads V2-V3 and subtle STE with Q-waves in lead aVL. Typically, RBBB presents with some STD and T-waves inversion in leads V1-V3 and can mask ST-T ischemic changes. Thus, in the right clinical context, any relative STE or upwards T-waves in these leads should be interpreted as possible acute ischemia [2]. In this case, the combination of clinical presentation suggestive of acute MI with the above ECG findings and the left ventricular wall motion abnormalities revealed by the echocardiogram is diagnostic of proximal LAD OMI. Moreover, according to the guidelines, this “non-STEMI (NSTEMI)” should be classified as very high risk because of patient’s acute heart failure symptoms due to ongoing myocardial ischemia [3]. Even so, the patient did not receive immediate reperfusion therapy. Unsurprisingly, Lupu et al. have found that only 6,4% of very high risk NSTEMI are taken for immediate coronary angiography [4].

STEMI/NSTEMI is a simplified and outdated classification of acute MI [5, 6] with STEMI criteria showing only 43,6% pooled sensitivity to detect acute coronary occlusion [7]. OMI paradigm has the potential to improve clinical outcomes of patients presenting with acute MI by reducing missed coronary occlusions. It expresses the pathophysiology of acute MI and incorporates clinical presentation with advanced ECG interpretation that can be facilitated by artificial intelligence ECG models, aided by echocardiography, CT imaging and troponin.

## Data Availability

No datasets were generated or analysed during the current study.

## Abbreviations

STEMI: ST elevation myocardial infarction
OMI: Occlusion myocardial infarction
MI: Myocardial infarction
RBBB: Right bundle branch block
LPFB: Left posterior fascicular block
ECG: Electrocardiogram
LAD: Left anterior descending coronary artery
STD: ST-segment depression
LCx: Left circumflex coronary artery
LAFB: Left anterior fascicular block
STE: ST-segment elevation
NSTEMI: Non-ST elevation myocardial infarction
